# Photocross-linking activity-based probes to capture the dynamics of ubiquitin RING E3 ligase interactions

**DOI:** 10.1042/BCJ20260213

**Published:** 2026-06-03

**Authors:** Sarah F. Chandler, Michael H. Tatham, Emma Branigan, Mark A. Nakasone, Nikolai Makukhin, Alessio Ciulli, Ronald T. Hay

**Affiliations:** 1 Division of Molecular, Cell and Developmental Biology, School of Life Sciences, University of Dundee, Dundee DD1 5EH, U.K.; 2 Centre for Targeted Protein Degradation, School of Life Sciences, University of Dundee, 1 James Lindsay Place, Dundee DD1 5JJ, U.K.; 3 Institute of Structural and Molecular Biology, School of Natural Sciences, Birkbeck, University of London, London WC1E 7HX, U.K; 4 Division of Biological Chemistry and Drug Discovery, School of Life Sciences, University of Dundee, James Black Centre, Dundee DD1 5EH, U.K.

**Keywords:** Activity-based Probes, cross-linking mass spectrometry, Structural Proteomics, Ubiquitin, ubiquitin ligases

## Abstract

Almost all cellular processes are influenced by ubiquitination. A large family of enzymes known as E3 ligases provides the specificity for ubiquitination, with the largest class among them, the Really Interesting New Gene (RING) E3s, comprising over 600 members in humans. RING E3s facilitate transfer of ubiquitin (Ub) to substrates by constraining the highly dynamic E2–Ub thioester linkage to be primed for attack from the substrate nucleophile. We have established a workflow that uses an N-maleimido diazirine (NMD) photoactivatable cross-linker attached to ubiquitin that, once stably linked to the active site of an E2, creates an activity-based probe (ABP) to monitor interactions with E3 ligases. Cross-linking mass spectrometry using the NMD-Ub-E2 ABP identified regions of interaction between ubiquitin and a selection of different RING E3s, which not only agreed with existing crystal structures, but was also used to evaluate *in silico* structural models of complexes yet to be resolved by conventional means. The cross-linking data also provided insight into domains of conformational flexibility which likely adopt multiple configurations in solution and which are challenging to monitor by other methods. NMD-Ub-E2 ABPs offer great potential to explore the ensemble conformations of E2–E3 complexes in solution and have scope for applications beyond the ubiquitin system.

## Introduction

Ubiquitination of target proteins requires three classes of enzymes: E1 ubiquitin-activating enzymes, E2 ubiquitin-conjugating enzymes, and E3 ubiquitin ligases [1]. Catalysed by the E1, ubiquitin first forms an ATP-dependent C-terminal adenylate prior to forming a thioester bond to a cysteine in the E1 with release of AMP. A transthiolation reaction delivers the ubiquitin to the catalytic cysteine of a bound E2 to generate a thioester-linked E2–Ub conjugate [2]. This is recruited to an E3 ligase to facilitate the transfer of ubiquitin to substrate [3]. The most common acceptor residue for ubiquitination is lysine, resulting in the formation of an isopeptide bond, although other residues, such as serine and threonine [4,5], can also be modified.

The mechanism of transfer of ubiquitin to substrates from E2–Ub is dependent on the type of E3 [6]. One group of ligases known as transthiolating E3s possess a catalytic cysteine and comprise ∼50 members, including Homologous to the E6-AP Carboxyl Terminus, RING-between-RING [7], and the recently discovered RING-Cys-Relay E3s [5]. The largest group of ∼600 consists of the adaptor class of E3s, which do not possess a catalytic cysteine and instead catalyse transfer of ubiquitin directly from the E2–Ub to substrate. They are characterised by so-called Really Interesting New Gene (RING) domains, which are zinc-coordinating scaffolds for the ubiquitin-loaded E2 [6]. Whilst some RING E3s are active as monomers, most form homodimers or heterodimers [8]. Some RING proteins, such as Rbx1 and Rbx2, mediate ubiquitination as components of multi-subunit complexes, such as the cullin-RING ligases [8], which all contain a cullin (Cul1, 2, 3, 4A, 4B, 5 or 7) scaffold protein to engage adaptor proteins that bind interchangeable substrate receptors.

RING E3 ligases conformationally constrain E2–Ub so the thioester linkage is primed for attack from the substrate nucleophile, typically the ε-amino group of a lysine residue [3]. To achieve this, the RING or RINGs contact both ubiquitin and E2, enforcing a ‘closed conformation’ [3,9–11]. In the absence of E3, the E2–Ub conjugates exist in ‘open conformations’ that are not favourable for transfer to substrate [12]. Understanding the ensemble of Ub–E2–E3 structures by conventional structural techniques is challenging due to their transient nature and conformational flexibility. Photocross-linking activity-based probes (ABPs) have promise in this area as they can trap transient conformations [13]. N-maleimido diazirine (NMD) is a particularly good candidate as it is heterobifunctional and can be linked to a specific site in a ‘probe’ protein through cysteine reaction with the maleimide-thiol, and once photoactivated, the carbene group reacts relatively non-specifically with proximal X-H bonds [14]. On this basis, we have established a workflow that uses an NMD-linked ubiquitin probe in complex with E2s that cross-links from the closed, active conformation to proximal E3 ligases. The identification by mass spectrometry (MS) of cross-linked peptides between ubiquitin and a variety of E3s not only confirmed known structures of E2–Ub–RING complexes but also identified transient complex formations.

## Results

### Design and characterisation of ABPs for RING E3 ligases

**Figure 1:**
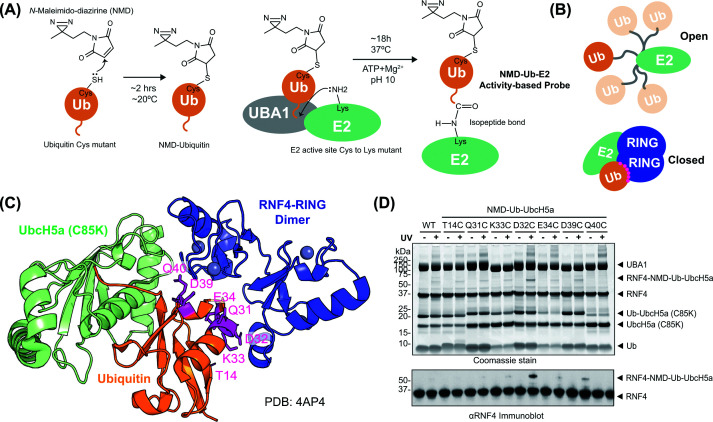
Design of an NMD-labelled ubiquitin-directed ABP to capture RING E3s (**A**) The photoreactive cross-linker NMD is linked to a cysteine introduced into ubiquitin by mutagenesis. NMD-Ub is conjugated to the active site of an E2 with the active site cysteine mutated to lysine, thereby forming a stable isopeptide-linked conjugate (NMD-Ub-E2) ABP. (**B**) Schematic representation of the open and closed conformations of E2 (green)-ubiquitin (orange) with E3 RINGs (dark blue). (**C**) Crystal structure of RNF4 RING dimer (blue) bound to ubiquitin (orange) conjugated to UbcH5a C85K mutant (pale green) in the closed conformation (PDB: 4AP4). Seven sites for photocross-linker coupling are indicated (magenta). (**D**) Photocross-linking of the seven different ubiquitin mutants in NMD-Ub-UbcH5a ABP with RNF4 + RING. Proteins were separated by SDS–PAGE and visualised with Coomassie blue staining (top) or immunoblotted for RNF4 (bottom).

Our E3-targeting ABP design accommodated two important considerations. Firstly, the Ub-E2 should be linked via an isopeptide bond rather than the unstable native thioester bond [3] (Figure 1A). Secondly, the photo-reactive cross-linker should be optimally positioned to capture interacting E3s when the Ub-E2 conjugate is in the closed conformation (Figure 1B) while also having minimal impact on ubiquitin structure and activity. Guided by the structure of the RNF4 RING dimer bound to Ub-UbcH5a [3], seven ubiquitin residues at the interface with the RNF4 RING domain (Figure 1C) were individually mutated to cysteine, and the proteins expressed and purified (Supplementary Figure S1A–C) for activity screening. Labelling each with NMD produced the expected mass increase of 179 Da (Supplementary Figure S1D). In multiple turnover RNF4-dependent 4xSUMO2 conjugation assays [15], all the NMD-modified forms of ubiquitin, apart from Q40C, were conjugated to substrate with comparable efficiency to wild-type, unmodified ubiquitin (Supplementary Figure S1E). This shows that neither the mutation nor the linked NMD adversely affected the ability of the modified ubiquitin variants to be activated by the E1, conjugated to the E2 and transferred to substrate by the E3. Likewise, all the NMD modified forms of ubiquitin, apart from Q40C, were efficiently loaded onto UbcH5a (C85K) in the final step to synthesise the NMD-Ub-UbcH5a ABPs (Supplementary Figure S1F). All ABPs were then tested for cross-linking to an RNF4 construct comprising full-length RNF4 linked to a second RING domain (RNF4 + RING). Individually, the probes were mixed with RNF4 + RING and half of each reaction was UV irradiated, while the other half was not. The products of both were analysed by SDS–PAGE (Figure 1D). Bands in stained gels corresponding to cross-linked NMD-Ub-UbCH5a-RNF4 + RING were apparent in the UV-irradiated samples for all the ubiquitin mutants except Q40C, with the D32C variant showing the highest cross-linking activity (Figure 1D). Therefore, while all ubiquitin variants except Q40C showed some potential for utility in cross-linking experiments, the D32C mutant was taken forward to test the concept.

**Figure 2:**
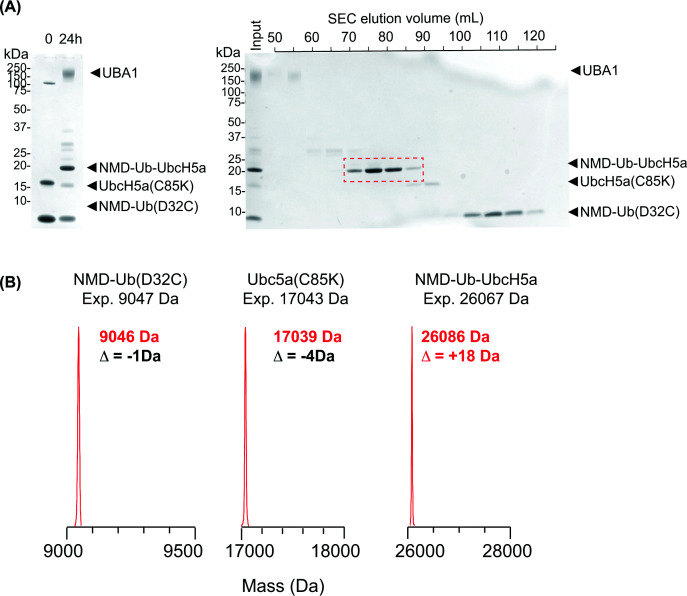
Preparation of the NMD-labelled ubiquitin-directed ABP (**A**) (Left) SDS–PAGE fractionation of conjugation reactions containing NMD-ubiquitin (D32C), UbcH5a (C85K), and UBA1. Reaction products were separated by size-exclusion chromatography (right), and fractions containing purified NMD-Ub-UbcH5a were pooled (red). (**B**) LC-MS analysis of NMD-ubiquitin (D32C), UbcH5a (C85K), and NMD-Ub-UbcH5a.

A large-scale synthesis and purification of this NMD-Ub (D32C)–UbcH5a (C85K) ABP (herein referred to as NMD-Ub-UbcH5a) was prepared (Figure 2A). Intact MS analysis (Figure 2B and Supplementary Figure S2A–C) showed that while the individual components, NMD-Ub (D32C) and UbcH5a (C85K), were of the expected masses prior to conjugation, the NMD-Ub-UbcH5a product was ∼18 Da larger than expected. Alkaline conditions can promote hydrolysis of maleimides [15–17] and efficient E1-mediated conjugation of ubiquitin to the E2 mutant requires high pH to deprotonate the acceptor lysine. Thus, to investigate if the reaction conditions used to generate the ABP caused the mass anomaly, we incubated NMD-labelled Ub (Q31C) at 37°C for 19 h under conjugation conditions and analysed the products by intact MS. These conditions did indeed generate a +18 Da species consistent with maleimide hydrolysis (Supplementary Figure S2D,E). While this was an unplanned consequence of the synthetic protocol, it brings benefits as hydrolysis of maleimide opens the ring to form maleamic acid, which prevents the retro-Michael reaction [18] to stabilise cross-links. Critically, the mass difference must be accounted for when searching for cross-links generated from these probes (see below).

### Photocross-linking with RNF4 confirms existing structures and identifies transient contacts between ubiquitin and non-RING domains

**Figure 3:**
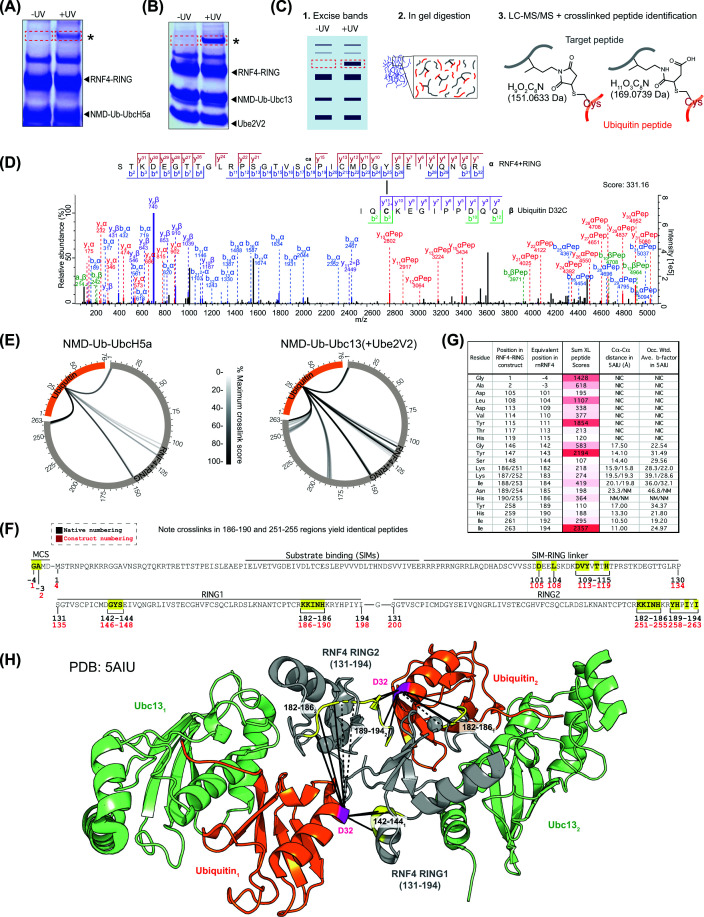
NMD–ubiquitin–E2-based photoreactive probes confirm the in-solution structure of RNF4 + RING bound by ubiquitin-loaded E2 (**A**,**B**) SDS–PAGE analysis of the products of photocross-linking (+UV) or control reactions (−UV) containing RNF4 + RING with either NMD-Ub-UbcH5a (**A**) or NMD-Ub-Ubc13 (**B**). *Asterisk marks position of UV-dependent species. (**C**) Workflow for identification of sites of cross-linking by MS. (**D**) Exemplar MS/MS spectrum for cross-linked peptide linking RNF4 Y143 to ubiquitin D32C. α peptide from RNF4 and β peptide from ubiquitin D32C (**E**) circular XiView [35] plots showing cross-links between RNF4 + RING (grey) and ubiquitin (orange) using ABPs containing UbcH5a (left) or Ubc13 (right). Each line represents a unique cross-linked pair of residues with lines shaded by cumulative score relative to the highest (100% black). (**F**) Cross-linked amino acids (yellow) from the RNF4 + RING construct comprising full-length RNF4 fused to a second RING domain. MCS – amino acids derived from the multiple-cloning site. (**G**) Corresponding distances between alpha carbons based on the published structure of Ub-Ubc13 bound to an RNF4 RING dimer (PDB: 5AIU). Sum of the scores of all cross-linked peptides that evidenced links between UbC32 and the specified E3 residue is shown along with occupancy-weighted b-factors from the structure. (**H**) Identified cross-links (solid lines) shown on the Ub-Ubc13-RNF4 + RING structure (5AIU). Cross-linked regions shown in yellow. Broken lines show cross-links to unmodeled regions.

To broaden the scope of the present study, we generated a second probe, NMD-Ub-Ubc13 ABP by conjugating NMD-labelled ubiquitin (D32C) to Ubc13 (C87K/K92A) (Supplementary Figure S3A). The two ABPs, NMD-Ub-UbcH5a and NMD-Ub-Ubc13 (combined with its heterodimeric partner, Ube2V2), were then tested for cross-linking to the RNF4 + RING construct. Both formed UV-dependent higher molecular weight adducts (Figure 3A,B), which were excised from the gel along with equivalent sections from the control lanes and tryptic peptides analysed by LC-MS/MS (Figure 3C). Protein intensity data confirmed that UV exposure increased amounts of the E2s, RNF4, and ubiquitin in the +UV gel sections (Supplementary Figure S3B,C), consistent with the formation of Ub–E2–RNF4 covalent cross-links. A significant challenge to the identification of covalently cross-linked peptides by MS is the combinatorial explosion in search space caused by considering all possible peptide pairs during peptide-spectrum matching [19]. While the diazirine end of the NMD cross-linker can react with all amino acids, by restricting one end (maleimide) to cysteines, the search space for NMD cross-links is considerably reduced. On this basis, the data were searched for both non-hydrolysed NMD (151.0633 Da – NMD-151) and the hydrolysed form (169.0739 Da – NMD-169) (Figure 3C). Only one cross-link involving the non-hydrolysed form remained after data filtering (see the ‘Materials and methods’ section) and was the only NMD-151 cross-link identified between Ub and an E3 among all the experiments described in the present study (Supplementary Datafile S1). We therefore focus the remainder of this report on NMD-169 cross-links.

Figure 3D shows an example of a high-scoring cross-linked peptide, in this case between ubiquitin C32 and Y147 from within RING1 in the RFN4 + RING construct, which shows compelling evidence for the covalent linking of the two peptides with NMD. Although subject to FDR filtering, all cross-links reported by MaxQuant are not of such high quality, which raises the issue of evaluation of large lists of reported cross-linked peptides. The crosslinkMsms.txt file often reports multiple MS/MS spectra for the same cross-linked amino-acid pair. This can be due to different combinations of differently cleaved peptides at each end of the cross-linker, different charge states of the same cross-linked peptides, and multiple sequencing events of the same charge state of the same peptide. These combine to give numerous pieces of evidence supporting the same cross-linked amino-acid pair. To allow a simple summary of the evidence supporting the different cross-links, we adopted an aggregated Andromeda score metric: the Andromeda scores for all MS/MS spectra evidencing the same cross-linked amino-acid pair were numerically summed to give a ‘Sum XL peptide score’ value. This metric is thus influenced by both the frequency and quality of the MS/MS spectra evidencing each cross-linked amino acid pair, with higher scores generally representing cross-links with multiple high-scoring MS/MS spectra. Any cross-linked amino-acid pairs with sum XL peptide scores less than 100 were rejected from our shortlists.

Based on these criteria, broad agreement was apparent for both probes (Figure 3E). Ubc13 provided the largest dataset of 21 cross-links, with many being within the two RINGs (Figure 3F). Mapping these onto the published structure of Ub-UBC13 bound to two RNF4 RINGs (PDB: 5AIU) showed most cross-linked residues were 10–20 Å away from ubiquitin D32, although His186 was not modelled in this structure (Figure 3G,H). While all these distances are beyond the nominal length of the NMD linker (8–10 Å), these Cα–Cα measurements do not account for side chain lengths or the precise point of reaction of the diazirine. Even so, some cross-linked residues, including Lys183, Ile184, and Asn185, appeared too far away from UbD32 (19.5, 20.1, and 23.3 Å, respectively) to react with the probe. It is likely that conformational flexibility of these regions of RNF4 explains this observation, as the b-factors of these residues are relatively high (Figure 3G), implying some in-solution conformations may bring these residues into closer proximity to UbD32 than shown in the crystal structure. Some high-scoring cross-links were also identified outside the RING domains (Figure 3F). Cross-links to the region linking the substrate-binding and RING domains indicate that these regions, at least transiently, contact the E2∼Ub. This is supported by single-molecule FRET experiments suggesting that the linker region, although disordered (Supplementary Figure S3D), may localise close to the RING [20] when bringing substrate towards the E2∼Ub to facilitate modification. The data for the UbcH5a-based probe was also consistent with the published crystal structure (PDB: 4AP4) in the resolved regions (Supplementary Figure S3E–G), albeit with fewer identified links.

### Mapping ubiquitin interactions with the heterodimeric RING E3 RNF2–BMI1

**Figure 4:**
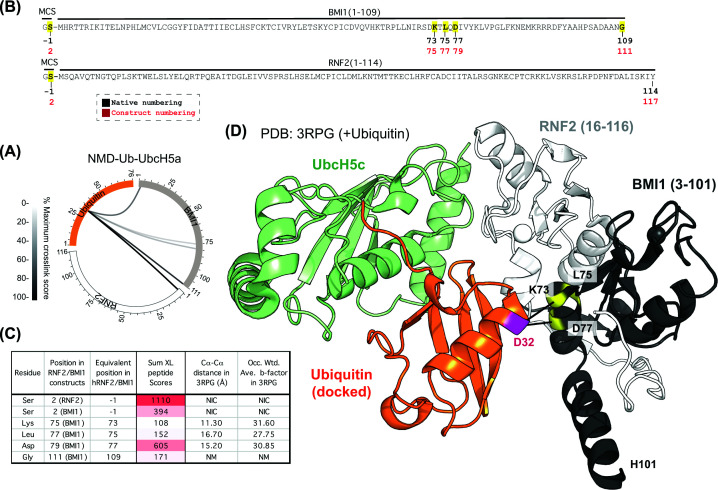
NMD-Ub-UbcH5a ABP captures the heterodimeric RING E3 RNF2/BMI1 (**A**) Circular XiView [35] plot showing cross-links between BMI1 (grey) or RNF2 (white) and ubiquitin (orange) using the NMD-Ub-UbcH5a ABP. Each line represents a unique cross-linked pair of residues with the line density showing the relative sum of the scores for each. Sequences and sites of cross-links (yellow) are shown in (**B**). MCS – amino acids derived from the multiple-cloning site. (**C**) Corresponding distances between alpha carbons based on the model in panel (D). Sum of the scores of all cross-linked peptides that evidenced links between UbC32 and the specified E3 residue is shown along with occupancy-weighted b-factors from the structure. (**D**) Published structure of UbcH5a-BMI1-RNF2 (PDB: 3RPG) with ubiquitin docked in the closed conformation.

Because we optimised our ABP with the RNF4 double RING construct, it was important to explore its utility with other E3 ligases. We first selected the heterodimeric E3 RNF2–BMI1, which has been structurally resolved in complex with UbcH5c but lacks ubiquitin [21]. We therefore undertook a cross-linking analysis using NMD-Ub-UbcH5a to interrogate a model generated by docking ubiquitin into this structure. As before, UV-exposed and control reactions were prepared and fractionated by SDS–PAGE (Supplementary Figure S4A). Background bands comigrating at the same position as the cross-linked product made them difficult to discern, but LC-MS/MS protein intensity data confirmed a UV-dependent increase in ubiquitin, RNF2, and BMI1 in the excised section (Supplementary Figure S4B). Only a single cross-link to RNF2 was identified, which was to an N-terminal residue derived from the plasmid polylinker (Figure 4A). BMI1 was also cross-linked close to the N-terminus (Figure 4A,B), as well as to the very C-terminal Gly109 (Figure 4A,B). A model generated by docking ubiquitin into the closed conformation in the existing structure suggests the remaining cross-links to residues Lys73, Leu75, and Asp77 in BMI1 were all <18 Å in distance from ubiquitin D32 (Figure 4C,D), consistent with this being a good representation of the complex in solution. An AlphaFold model based on the RNF2, BMI1 ubiquitin and UbcH5a constructs used in our cross-linking experiment compared favourably to the docked ubiquitin structure (Supplementary Figure S4C), and confirmed the N-terminal regions of RNF2 and BMI1 are likely to be relatively flexible (low pLDDT scores), potentially explaining their cross-linking over apparently long distances. Gly109 in BMI1 was also missing from the crystal structure but simple extension of the α-helix truncated in the crystal structure (Figure 4D) would take it well beyond the range of the cross-linker. However, the AlphaFold model tentatively suggests that Gly109 may sit at the end of a helical turn (Supplementary Figure S4C), putting it only 12.2 Å from UbC32.

### RNF38 photocross-linking with NMD-Ub-UbcH5a confirms the existing structure and identifies mobile regions of the ligase

**Figure 5:**
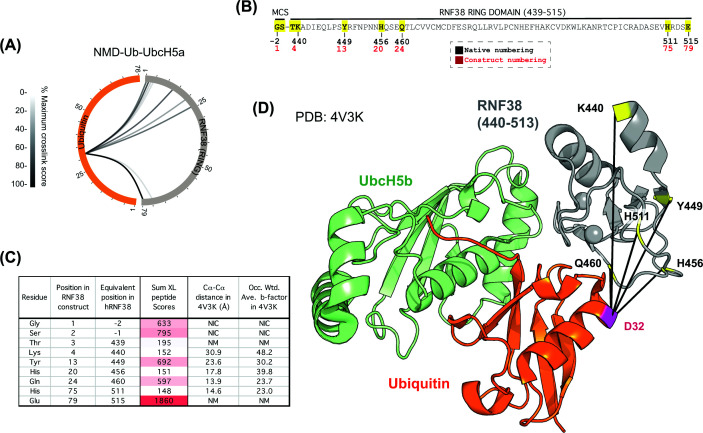
NMD-Ub-UbcH5a ABP captures the monomeric RING E3 RNF38 (**A**) Circular XiView [35] plot showing cross-links between RNF38 (grey) and ubiquitin (orange) using the NMD-Ub-UbcH5a ABP. (**B**) Amino acid sequence of the RNF38 (439-515) construct with cross-linked residues (yellow). MCS – amino acids derived from the multiple-cloning site. Distances between α-carbons calculated (**C**) based on the Ub-UbcH5b-RNF38 structure (PDB: 4V3K). Sum of the scores of all cross-linked peptides that evidenced links between UbC32 and the specified E3 residue is shown along with occupancy-weighted b-factors from the structure. (**D**) PDB: 4V3K with identified cross-links shown.

We next tested the monomeric RING ligase RNF38, which has been resolved by crystallography in complex with Ub-UbcH5b [22]. We performed a photocross-linking experiment using the NMD-Ub-UbcH5a probe with the RING domain of RNF38 (residues 439–515) (Supplementary Figure S5A,B) and found nine high-scoring cross-links (Figure 5A–C). Again, cross-links to N- and C-terminal amino acids were apparent, although they were not mapped in the published structure (Figure 5D). Of those that were resolved, H456, Q460, and H511 were <18 Å from D32 of ubiquitin. K440 and Y449 were 30.9 and 23.6 Å away, although, as was the case for RNF4, the high b-factors of these residues imply structural flexibility (Supplementary Figure S5C), potentially explaining the apparent distance conflicts and reinforcing the observation that cross-links can form with apparently distant residues if the associated domains are relatively flexible.

### Cross-linking evidence that Ub-UbcH5a can bind a symmetrical CHIP dimer

**Figure 6:**
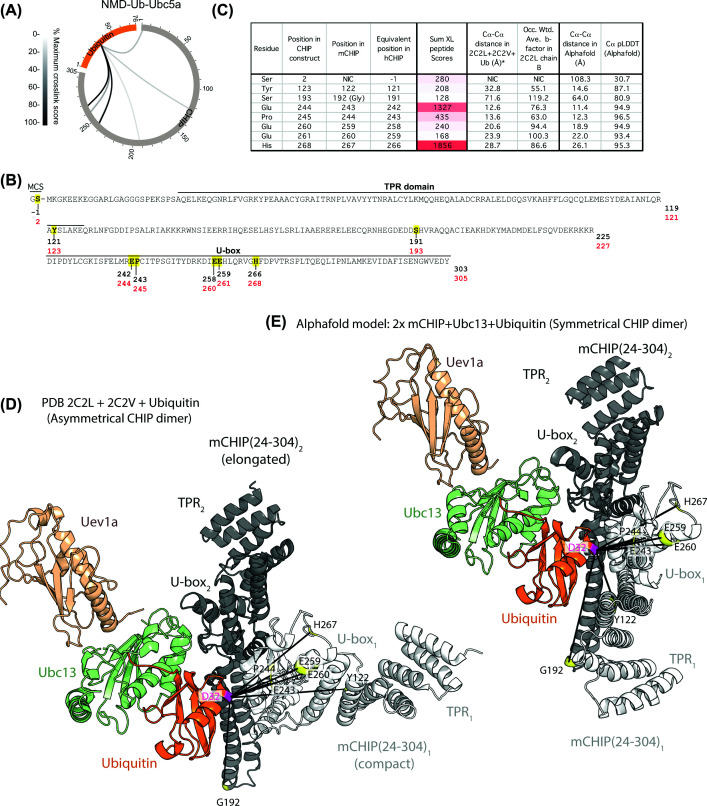
Cross-linking between NMD-Ub-UbcH5a and CHIP supports a symmetrical dimer binding arrangement (**A**) XiView [35] summary for cross-links between NMD-Ub-UbcH5a and hCHIP. (**B**) Sequence of CHIP used in the present study with yellow residues showing sites of cross-linking. (**C**) Distances measured between α-carbons of ubiquitin C32 and cross-linked residues from CHIP according to the models shown in panels (D,E). Sum of the scores of all cross-linked peptides that evidenced links between UbC32 and the specified E3 residue is shown along with occupancy-weighted b-factors from the structure or pLDDT scores from AlphaFold. (**D**) Model generated by docking Ubc13/Uev1a from PDB: 2C2V onto the crystal structure of the apo full-length mouse CHIP asymmetrical dimer PDB: 2C2L by aligning U-box domains, with a single ubiquitin (D32C) docked in the closed conformation. (**E**) AlphaFold model of two copies of mouse CHIP with one each of Ubc13 and ubiquitin.

We have tested our ABPs with various RING-containing E3s. To determine if we could employ them to analyse interactions with non-RING E3s, we tested the U-box E3 ligase CHIP. U-boxes have similar folds to RING domains but lack zinc ions and are instead stabilised by hydrogen bonds and salt bridges [7]. Full-length human CHIP was cross-linked to the NMD-Ub-UbcH5a probe (Supplementary Figure S6A,B), resulting in identification of eight cross-links (Figure 6A,B). Consistent with the other E3s tested, the N-terminus of CHIP formed a cross-link to UbC32. Five cross-links mapped to the U-box domain (E242, P243, E258, E259, and H266), one to the TPR domain (Y121), and one to the protomer crossover helix (S191). Although no human CHIP-E2–Ub complex structure is available, a crystal structure of the apo mouse CHIP showed the E3 in an asymmetrical homodimer composed of an ‘elongated’ protomer possessing a long linear helical domain projecting away from the U-box domain, and a ‘compact’ protomer where the equivalent helical domain contains a ‘break’ halfway along its length, causing a bend [23]. This break twists the TPR and U-box domains out of alignment, meaning only one Ub-E2 thioester can bind to the CHIP dimer, and does so with the ‘elongated’ protomer. To assess our cross-linking data, we modelled a Uev1a-Ub-Ubc13-mCHIP dimer by combining the two published structures of Uev1a-Ubc13 + mCHIP (U-Box domain) (PDB: 2C2V) with the full-length CHIP dimer (PDB: 2C2L) and docked ubiquitin into the closed conformation on Ubc13 (Figure 6D). In this composite model most of the interaction between the Ub and Ubc13 occurs with the U-box domain of the ‘elongated’ CHIP protomer. The cross-links identified were closer to the U-box of the other ‘compact’ CHIP protomer (Figure 6D). Cross-links to E243 and P244 in the U-box α-helix are 12.6 and 13.6 Å, respectively, from UbD32 (Figure 6C,D). However, cross-links to E259 and E260, also in a helical region, are both over 20 Å away from ubiquitin D32 (Figure 6C,D). Furthermore, H267 (predicted to be in a disordered U-box region) and Y122 in a TPR helix are both ∼30 Å away from UbD32. Due to relatively low resolution of the mCHIP dimer structure, flexibility could not be assessed by b-factor, so an AlphaFold model was generated using two copies of mCHIP (24–304) and one each of Uev1a, Ubc13 and ubiquitin, and the pLDDT was used as a proxy for disorder propensity (Figure 6C). While the positioning of Ub-Ubc13 and one CHIP protomer was consistent with the composite structure, AlphaFold predicted a symmetrical dimer, rather than an asymmetric form (Figure 6D,E). In this symmetrical dimer the U-box and TPR domains are considerably closer to ubiquitin D32 than in the asymmetric dimer model, and all predicted cross-linker distances are shorter (Figure 6C). The most striking difference is Y122, which is over 18 Å closer to ubiquitin D32 in the symmetrical model. These data suggest that a symmetrical CHIP dimer may exist, at least temporarily in solution, which could bind two copies of ubiquitin-loaded E2s.

A second AlphaFold model was generated using two copies of each of the proteins used in the cross-linking study: hCHIP (1–303), UbcH5a, and ubiquitin (Supplementary Figure S6C–E). This confirmed that the N-terminal cross-link is likely to be due to high mobility and intrinsic disorder of the CHIP N-terminal domain (Supplementary Figure S6C). It also showed that the TPR and U-box domains are confidently modelled and support a 2:2 (CHIP:Ub-E2) stoichiometry complex (Supplementary Figure S6D). This is consistent with a recent cryo-EM study using an Fab that interacts with the E2 binding site in the CHIP U-box [24], which found CHIP can adopt a symmetrical structure. The symmetrical structure is also supported by MD simulations [25] and the structure of a TPR-depleted version of CHIP [26].

## Discussion

Understanding transient conformations of protein complexes poses a challenge to traditional structural techniques. ABPs have great potential in this endeavour, as they can provide information on the broader ensemble of protein complex formations in solution. While some ABPs have been reported for ubiquitin RING E3s [27] and SUMO E3s [28], they have not been used to provide detailed structural mapping of these complexes. Therefore, there is potential to combine ABPs with cross-linking MS to provide more detailed information on Ub–E2–E3 complexes in solution. We have developed a photocross-linking ABP based on an E2 enzyme linked via an isopeptide bond to ubiquitin carrying an NMD cross-linker. We have used this in conjunction with cross-linking MS to precisely map sites of interaction with multiple RING E3 types. Cross-links were identified that were consistent with known crystal structures or models generated from crystal structures, validating this approach as a reliable method for probing structures.

Despite this, many well-evidenced cross-links were not apparently consistent with available structures. Notably, in all cross-linking experiments, residues close to the N-termini of the E3 constructs formed cross-links with the ubiquitin probe. Most likely, these cross-links are created by virtue of occasional proximity of flexible domains in a fraction of the ensemble of conformations in solution, rather than fixed proximity [29,30]. Even within the structured RING domains of the E3s, cross-links were found that, according to the published crystal structures, were too distant to be linked via NMD. In these cases, cross-links were localised to regions of elevated b-factor (Supplementary Figure S6F), and therefore, these apparent distance violations are likely to indicate regions of the E3 that adopt multiple conformations in solution. These observations support a more general conclusion that high-scoring cross-links can be broadly categorised into two groups: (i) residues regularly in close proximity from relatively static domains, or (ii) residues occasionally in close proximity in relatively flexible domains. These cannot be differentiated by MS alone, and so this emphasises the difficulty in interpreting cross-links in the absence of existing structures. To assist in cross-link interpretation, we used the structure-prediction algorithm AlphaFold. AlphaFold reports pLDDT (Predicted Local Distance Difference Test) values for all residues in a model, which represents confidence in the reported residue position. We generated multiple Ub–E2–E3 AlphaFold models based on the proteins used in the cross-linking experiments (Supplementary Figures S3D, S4C, and S5C and Supplementary Datafile S2) and observed a strong inverse correlation between Cα–Cα distance and pLDDT (Supplementary Figure S6G). Therefore, similarly to the crystal structure b-factors (Supplementary Figure S6F), longer Cα–Cα distances are indicative of flexible or disordered domains that may adopt conformations not presented in the models. For the NMD cross-linker specifically, our data suggest Cα–Cα distances below 40 Å in combination with AlphaFold pLDDT scores greater than 70 (supplementary Figure S6G) are indicative of residues close to the probe in most conformations within the ensemble. Longer cross-links with lower pLDDT scores indicate residues that are only close in a minority of the total ensemble of conformations (Supplementary Figure S6G). However, these are not fixed rules and cross-links violating this relationship may also be informative. For example, S191 in hCHIP was found cross-linked to UbC32 over 70 Å away in the static model (Supplementary Figure S6E), but with a relatively confident pLDDT score of 81. S191 is at the very apex of the helical crossover region of CHIP and is significantly different between the elongated and compact forms. But even in the compact conformation, S191 is still >60 Å from UbC32, suggesting that this cross-link may form when CHIP is in a conformation not shown by either the symmetrical or asymmetrical dimer, perhaps during the process of flipping between the two, when MD simulations suggest that the helix unwinds [25].

In summary, this work demonstrates the successful application of NMD–ABPs in exploring the dynamic nature of Ub–E3–E3 complexes in solution. We show that combining cross-linking MS with *in silico* structural modelling provides details of molecular dynamics that are not apparent in structures resolved by traditional methods. While we based our studies on ubiquitin (D32C), it is likely that cross-linker positioning at other sites in ubiquitin would give additional useful information. We also expect that the NMD-based cross-linking pipeline described here will have utility beyond the ubiquitin system.

## Materials and methods

### Expression and purification of RNF4 + RING, ubiquitin, UbcH5a (C85K), Ubc13 (C87K/K92A), and Ube2V2

Proteins were expressed in *Escherichia coli* BL21 (DE3). Starter cultures (10–25 ml) of LB medium supplemented with appropriate antibiotics were inoculated from freshly streaked LB agar plates and incubated overnight at 37°C with shaking at 220 rpm. Five millilitres of overnight culture was used to inoculate 500 ml of LB medium containing appropriate antibiotic in a 2 L flask. Cultures were grown at 37°C with shaking at 220 rpm until an OD_600_ ∼0.6–0.8 was reached. Cultures were cooled on ice for 10–20 min prior to induction with 100 μM IPTG at 20°C with shaking at 220 rpm for 17–19 h. Bacterial cell pellets were resuspended in lysis buffer consisting of 50 mM Tris, 250 mM NaCl, and 10 mM imidazole, adjusted to pH 7.5, and supplemented with EDTA-free complete protease inhibitor cocktail (Roche), using 40 ml buffer per 0.5 L of cell culture. Cell suspensions were lysed by sonication (Digital Sonifier, Branson), and insoluble material was removed by centrifugation (27 200 × ***g***, 45 min, 4°C). The clarified lysate was filtered through a 0.2 μm filter and loaded onto a Ni–NTA agarose column (Qiagen) using 1 ml resin per 0.5 L culture, pre-equilibrated with binding buffer containing 50 mM Tris, 250 mM NaCl, and 10 mM imidazole, pH 7.5. The column was washed sequentially with approximately 8 column volumes of binding buffer followed by 6–8 column volumes of washing buffer containing 50 mM Tris, 250 mM NaCl, and 30 mM imidazole, pH 7.5. Bound proteins were eluted using 50 mM Tris, 150 mM NaCl, 150 mM imidazole, and 0.5 mM TCEP, pH 7.5. Pooled Ni–NTA elution fractions were dialysed overnight at 4°C against buffer containing 50 mM Tris, 150 mM NaCl, and 0.5 mM TCEP after addition of 1 mg TEV protease per 10 mg of 6His-tagged ubiquitin or 1 mg TEV protease per 50 mg for all other 6His-tagged proteins. For incomplete cleavages, additional TEV protease was added, and samples were incubated at room temperature for approximately 4 h. After cleavage, imidazole was added to a final concentration of 10 mM, and samples were centrifuged (3900 × ***g***, 15 min, 4°C) to remove precipitated material. The clarified supernatant was applied to a Ni–NTA agarose column pre-equilibrated with buffer containing 50 mM Tris, 150 mM NaCl, 10 mM imidazole, and 0.5 mM TCEP, pH 7.5. The flow-through fraction was collected and if homogeneous, samples were dialysed against 50 mM Tris, 150 mM NaCl, and 0.5 mM TCEP, pH 7.5, and concentrated to approximately 500 μM using Vivaspin centrifugal concentrators (Sartorius; 3–10 kDa MWCO) at 3,000 g and 4°C, and aliquots stored at −80°C. Where necessary, further purification by size-exclusion chromatography was performed. SDS–PAGE followed by Coomassie blue staining was used to assess fractions and those containing homogenous protein were pooled, concentrated, flash-frozen in liquid nitrogen, and stored at −80°C.

### Expression and purification of 6His-hUBA1

Expression of 6His-hUBA1 in *Escherichia coli* BL21 (DE3) cells was performed at large scale due to typically low expression levels. Cultures were supplemented with 1 mM MgSO_4_ per litre to support high-density growth and with ampicillin (100 μg/ml). Starter cultures were inoculated at approximately 10 ml per litre of LB medium. Cultures were grown at 37°C until an OD_600_ of ∼0.8 was reached (approximately 6–8 h), after which the temperature was reduced to 16°C. Protein expression was induced with 0.25–0.4 mM IPTG for an additional 16–20 h. Harvested cells were resuspended in 50 mM phosphate buffer, 500 mM NaCl, and 40 mM imidazole, adjusted to pH 7.5. Approximately 24 L of culture yielded ∼300 ml of cell suspension. Cells were passed through a Conti cloth filter and lysed by high-pressure cell disruption (Avestin). MgCl_2_ was added to a final concentration of 1 mM together with DNase (0.5 mg/ml). Lysates were clarified by centrifugation (19 000 rpm, 45 min, 4°C; 45Ti rotor) and filtered through a 0.45 μm syringe filter prior to Ni–NTA chromatography. Clarified lysates were loaded onto a 5 ml HisTrap column pre-equilibrated in the same buffer as the protein at a flow rate of 2.5–4.0 ml/min. After loading, the column was washed with 25 column volumes of the same buffer and hUBA1 was eluted using a 50 mM phosphate buffer, 500 mM NaCl, and 350 mM imidazole, pH 7.5. Eluted hUBA1 was concentrated using a 30 kDa MWCO Amicon centrifugal filter unit and diluted in 10 × 50 mM Tris and 5 mM β-mercaptoethanol, pH 8.0, to reduce the salt concentration to ∼50 mM. The sample was loaded onto a 5 ml HiTrap Q-HP anion exchange column (Cytiva) and washed with 5 column volumes of Anion Buffer A. Proteins were eluted using a 0–50% gradient of 50 mM Tris, 1 M NaCl, and 5 mM β-mercaptoethanol, pH 8.0, over 40–60 column volumes. Fractions containing hUBA1 were concentrated and further purified by size-exclusion chromatography on a Superdex 200 16/600 column (Cytiva) equilibrated in 25 mM Tris, 300 mM NaCl, and 0.5 mM TCEP, pH 8.0. Homogeneous hUBA1 was assessed by SDS–PAGE analysis and Coomassie blue staining. Aliquots were stored at −80°C.

### Expression and purification of untagged ubiquitin

Untagged ubiquitin variants expressed from the pET24a vector were purified using ammonium sulphate precipitation followed by ion exchange and size-exclusion chromatography. Induced bacterial cell pellets were resuspended in a lysis buffer containing 50 mM Tris, 250 mM NaCl, and 0.5 mM TCEP, adjusted to pH 7.5, and supplemented with EDTA-free complete protease inhibitor cocktail (Roche). Cells were lysed by high-pressure cell disruption (Avestin), and insoluble material was removed by centrifugation (27 200 × ***g***, 45 min, 4°C). The clarified lysate was filtered through a 0.2 μm filter, and solid ammonium sulphate was added to 48% saturation for 1 h at room temperature, followed by centrifugation (4500 rpm, 20 min, room temperature). To precipitate ubiquitin, solid ammonium sulphate was added to the supernatant to 90% saturation and incubated for 1 h at room temperature. The supernatant was discarded, and the pellet resuspended in 2 L of low-salt buffer containing 10 mM ammonium acetate and 0.5 mM TCEP, pH 4.5. Ion exchange chromatography was performed using an ÄKTA Pure system (Cytiva) with a HiTrap SP HP 5 ml column pre-equilibrated in low-salt buffer. After washing with 10 column volumes of low-salt buffer, proteins were eluted using a linear gradient from low-salt to high-salt buffer containing 10 mM ammonium acetate, 0.5 mM TCEP, and 1 M NaCl, pH 4.5. The column was washed with 10 column volumes of high-salt buffer. Fractions containing homogenous ubiquitin were identified by SDS–PAGE, pooled, and concentrated to approximately 10 mg/ml using Vivaspin centrifugal concentrators (Sartorius; 3 kDa MWCO) at 3000 × ***g*** and 4°C. Samples were further purified by size-exclusion chromatography on a HiLoad 26/600 Superdex 75 pg column (GE Healthcare) equilibrated in a buffer containing 50 mM Tris, 150 mM NaCl, and 0.5 mM TCEP, pH 7.5. Fractions containing homogenous ubiquitin were pooled, concentrated to approximately 500 μM, aliquoted, flash-frozen in liquid nitrogen, and stored at −80°C.

### Expression and purification of RNF38 RING and RNF2/BMI1 heterodimer

pGEX4T1 expressing GST-TEV-RNF38 (isoform 1) residues 439–515 [22] was transformed into *Escherichia coli* BL21 (DE3) Rosetta 2 pLysS (Novagen) chemically competent cells. pGEX4T1 expressing GST-TEV-RNF2 residues 1–114 and pRSFDuet1 expressing 6His-TEV-BMI1 residues 1–109 [31] were co-transformed into the same cells and selected using ampicillin and kanamycin. Twelve litres of cultures were grown in Luria–Bertani (LB) medium at 37°C to an OD_600_ of ∼0.8 supplemented with 1 mM MgSO_4_, 200 μM ZnSO_4_ was added before induction, and cells were induced with 0.25 mM IPTG at 20°C for 12–16 h. Cell pellets were harvested by centrifugation at 3500 rpm for 30 min at 4°C. The pellets were resuspended in 25 mM Tris–HCl, 500 mM NaCl, 5 mM DTT, pH 7.5 with 1 mM MgCl_2_ and DNase I. Lysis was performed at 12 000 psi using a microfluidiser operating at 4°C. The lysates were cleared by high-speed centrifugation at 32 000 × ***g*** for 45 min at 4°C. Then supernatant was filtered through a 0.45-μm syringe filter and loaded on a 20 ml GST-Trap HP column (Cytiva) at 2 ml/min. Once loaded, the UV absorbance reached baseline over the 20 column volumes (cv) wash. GST proteins were eluted over 5 cv in 25 mM Tris–HCl, 500 mM NaCl, 2 mM TCEP, 20 mM reduced L-glutathione, pH 7.8. The elution was dialysed overnight in 3.5 MWCO Snakeskin (ThermoFisher) at 4°C in 25 mM HEPES, 350 mM NaCl, 1 mM TCEP, pH 7.5 buffer. To ensure a robust heterodimer, GST-TEV-RNF2/6His-TEV-BMI1 was subjected to a loading and elution buffer (25 mM HEPES, 350 mM NaCl, 350 mM imidazole, 1 mM TCEP, pH 7.5) on a 10 ml His-Trap HP column, then dialysed with TEV in a separate step. TEV protease and residual GST were removed using a pass-back step through GST-Trap and His-Trap columns, and the flowthrough was concentrated using 3500 Da MWCO Amicon centrifugal units. The final purification step was size-exclusion chromatography using a 26/600 Superdex 75 column (Cytiva) pre-equilibrated in 25 mM HEPES, 200 mM NaCl, 1 mM TCEP, pH 7.5. Protein purity was assessed using SDS–PAGE and aliquots stored at −80°C.

### Expression and purification of full-length CHIP

pRSF-Duet1 containing human full-length 6His-TEV-CHIP [32] was transformed into *Escherichia coli* BL21 (DE3) Rosetta (Novagen) cells and selected with kanamycin. Twelve litres of cultures were grown in LB medium at 37°C to an OD_600_ of ∼0.8 supplemented with 1 mM MgSO_4_ and cells were induced with 0.25 mM IPTG at 20°C for 12–16 h. Cell pellets were harvested by centrifugation at 3500 rpm for 30 min at 4°C. The pellets were resuspended in His buffer A (25 mM HEPES, 500 mM NaCl, 2 mM TCEP, pH 7.5) with 1 mM MgCl_2_ and DNase I. Lysis was performed at 12 000 psi using a microfluidiser operating at 4°C. The lysates were cleared by high-speed centrifugation at 32 000 × ***g*** for 45 min at 4°C. Then supernatant was filtered through a 0.45-μm syringe filter and loaded on a 10 ml His-Trap HP column (Cytiva) at 2 ml/min. Once loaded, the UV absorbance reached baseline over the 30 cv wash. 6His-TEV-CHIP was eluted over 6 cv in His buffer B (25 mM HEPES, 500 mM NaCl, 350 mM imidazole, 1 mM TCEP, pH 7.5). The elution was taken for overnight dialysis in 3.5 MWCO Snakeskin (ThermoFisher) at 4°C in 25 mM HEPES, 350 mM NaCl, 1 mM TCEP, pH 7.5 buffer with addition of TEV protease to 1:100 (w/w). TEV protease was removed using a pass-back step through a 10 ml His-Trap column and the flow-through was concentrated using 10 000 Da MWCO Amicon centrifugal unit. This was loaded on a 26/600 Superdex 200 column (Cytiva) pre-equilibrated in 25 mM HEPES, 250 mM NaCl, 1 mM TCEP, pH 7.5 buffer. Fractions were checked for purity by SDS–PAGE, concentrated, and aliquots stored at −80°C.

### Preparation of NMD-labelled ubiquitin

NMD [14] stocks were prepared at 10 mM in anhydrous DMSO and stored in 50 μl aliquots at −20°C. Ubiquitin cysteine variants were buffer-exchanged into degassed buffer containing 50 mM Tris and 150 mM NaCl, pH 7.0, using Centri Pure Zetadex-25 gel filtration columns (Generon). Labelling reactions were performed at room temperature for 2 h using a five-fold molar excess of NMD relative to protein at a protein concentration of 200 μM. Excess reagent was removed by gel filtration using Centri Pure Zetadex-25 columns equilibrated in buffer containing 50 mM Tris, 150 mM NaCl, and 0.5 mM TCEP, pH 7.5. All buffers were degassed, all labelling reactions and labelled proteins were protected from light, and all products were analysed by intact LC-MS.

### NMD-labelled ubiquitin multiple turnover assay

Ubiquitination reactions were carried out by incubating a mixture containing 0.1 μM 6His-UBA1, 0.5 μM UbcH5a, 0.55 μM RNF4, 5.5 μM 4xSUMO-2, 20 μM ubiquitin, 50 mM Tris, 150 mM NaCl, 5 mM MgCl_2_, 0.5 mM TCEP, and 0.1% NP-40 at room temperature. The reaction was initiated by adding 3 mM ATP and stopped by addition of reducing SDS–PAGE loading buffer to 2× concentration. Time points were collected at intervals (0, 2, 5, 10, 20, 40, 60, and 100 min), the zero-time point was taken before ATP addition. Samples were incubated at 95°C for 5 min and analysed using SDS–PAGE on a 10% NuPAGE Bis-Tris polyacrylamide gel with MES buffer, followed by Coomassie Blue staining.

### Preparation of the NMD-Ub-UbcH5a and NMD-Ub-Ubc13 ABPs

UbcH5a (C85K) (25 μM) was incubated with NMD-labelled ubiquitin D32C (100 μM) and 6His–Uba1 (1 μM) at 37°C for 24 h in conjugation buffer (50 mM Tris pH 10.0, 5 mM MgCl_2_, 1 mM TCEP). ATP (3 mM) was added to initiate the reaction. Homogenous NMD-Ub-E2 was collected using a HiLoad 16/600 Superdex 75 pg column (GE Healthcare) pre-equilibrated with 20 mM HEPES, 150 mM NaCl, 1.0 mM TCEP, pH 7.5. Hamilton syringe (5 ml) was used to inject a 3 ml reaction onto the column. The flow rate was 0.5 ml/min, fractions were 0.5 ml, and homogenous NMD-Ub-E2 was confirmed by SDS–PAGE analysis with Coomassie blue staining. The purified NMD-Ub-E2 was concentrated (Cytiva, Vivaspin, MW cut-off 10 kDa) and stored at −80°C. For the Ubc13-based ABP, Ubc13 C87K, K92A (50 μM) was incubated with NMD-labelled ubiquitin D32C (60 μM) and 6His–Uba1 (0.8 μM) at 37°C for 21 h in conjugation buffer (50 mM Tris pH 10.0, 150 mM NaCl, 5 mM MgCl_2_, 0.5 mM TCEP). Three millimolar ATP was added to initiate the reaction. Homogenous NMD-Ub-E2 was collected using a HiLoad 16/600 Superdex 75 pg column (GE Healthcare) pre-equilibrated with 20 mM HEPES, 150 mM NaCl, 1.0 mM TCEP, pH 7.5. Hamilton syringe (5 ml) was used to inject a 3 ml reaction onto the column. The flow rate was 0.5 ml/min, fractions were 0.5 ml, and homogenous NMD-Ub-E2 was confirmed by SDS–PAGE analysis with Coomassie blue staining. Purified NMD-Ub-E2 was concentrated (Cytiva, Vivaspin, MW cut-off 10 kDa) and stored at −80°C.

### Photocross-linking of NMD-Ub-UbcH5a or Ubc13 ABPs

Photocross-linking reactions (40–100 μl) were performed with NMD-Ub-UbcH5a or NMD-Ub-Ubc13/Ube2V2 (∼10 μM) and target protein (10 μM) in reaction buffer (20 mM HEPES, pH 7.5, 150 mM NaCl, 1 mM TCEP). Samples were divided into two portions. One portion was irradiated at 365 nm ∼15 cm away from a 365 nm LED lamp (UHP-T-365-MP, Prizmatix) in an 18-well glass-bottom plate (Ibidi) on an ice-cold metal block for 10 min and the other portion was preserved in the dark on ice. Samples were resolved by SDS–PAGE, using 10% NuPAGE Bis-Tris polyacrylamide 1.5 mM gels to load sample volumes above 20 μl. Proteins were visualised by Coomassie staining.

### MS sample preparation by in-gel tryptic digestion

Protein samples were diluted in NuPAGE LDS Sample Buffer (Invitrogen) to 1.2× operating concentration, separated by SDS–PAGE, and visualised using filtered Coomassie blue in a sterile dish. The protein bands of interest were cut out of the gel into ∼1 mm cubes. The gel pieces were de-stained overnight in 50 mM ammonium bicarbonate (ABC) and 50% acetonitrile (ACN). Proteins were reduced with 10 mM DTT for 30 min and alkylated in 50 mM iodoacetamide (VWR) in the dark for 30 min. Gel pieces were washed with 100 mM ABC and then 20 mM ABC, 50% ACN before being dehydrated with 100% ACN for 5 min. Gel pieces were dried in a fume hood for 15 min. Trypsin (Thermo) was added (1:100 w:w) according to estimated protein amounts in each band. Trypsin was diluted in 20 mM ABC, 9% ACN, and added to the gel pieces in a volume that just covered the pieces (rehydration volume, RV). Gel pieces were incubated for 16 h at 37°C. To extract the peptides, the RV of 100% ACN was added to the gel pieces, followed by RV 5% formic acid, 50% ACN. Pieces were dehydrated again by adding RV 100% ACN. Peptides were dried down, and pellets were resuspended in 0.1% trifluoroacetic acid (TFA) 0.5% acetic acid and submitted for MS analysis. Gel pieces in suspension were shaken at 1400 rpm at 20°C during all steps.

### LC-MS/MS peptide analysis

Samples were analysed on a Thermo Fisher Scientific Lumos Tribrid mass spectrometer coupled with a Thermo Dionex Ultimate 3000 RSLC HPLC. The buffers used for HPLC were 0.1% formic acid as buffer A and 80% ACN with 0.08% formic acid as buffer B. Trap column Acclaim PepMap 100 (C5, 100 μM × 2 cm) was used before the main column for sample concentration and cleanup. The peptide samples were loaded onto the trap column using a loading pump with 3% ACN and 0.1% TFA at a flow rate of 5 μl/min. The main column used was EASY-Spray column (C18, 2 μm, 75 μm × 50 cm) with a nano-electrospray emitter built in. The flow rate of 300 nl/min was maintained throughout the run. Peptides were separated with a 90 min gradient as detailed within raw data files (see the ‘Data availability’ section for further details). The separated peptides were analysed on the mass spectrometer with the following settings. Spray voltage was 2 kV, RF lens level was 30%, and ion transfer tube temperature was 275°C. The mass spectrometer was operated in data-dependent mode with 2 s cycle time. The full scan was performed in the range of 375 to 1500 mass/charge ratio (m/z) at a nominal resolution of 120 000 at 200 m/z and automatic gain control (AGC) was set to 400 000 with a custom maximum injection time of 50 ms. This was followed by the selection of the most intense ions above an intensity threshold of 20 000 for higher-energy collision dissociation fragmentation, with normalised collision energy set to 30. MS2 scans were acquired for charge states 2 to 7 using an isolation width of 1.6 m/z. MS2 scans were done at resolution 60K using an AGC target of 500 000 and a maximum fill time of 500 ms. Dynamic exclusion was set to 30 s.

### Identification of NMD-linked peptides

Data analysis was performed with MaxQuant version 2.4.0.0. Default settings were used with a few exceptions: A database of all the recombinant proteins included in the cross-linking assays was used. Digestion was set to trypsin/P (ignoring lysines and arginine N-terminal to prolines) with a maximum of 8 missed cleavages. Match between runs was enabled. Prior to running the search, two new cross-linkers were added to MaxQuant: hydrolysed NMD named ‘NMD169’, linked to composition H(11)O(3)C(8)N, mass 169.0738932246 Da. NMD named ‘NMD151’: Linked composition H(9)O(2)C(8)N, mass 151.0633285383 Da. Specificity 1 was C; position in peptide 1 was set to anywhere. Protein N-term 1 and C-term 1 were selected. Specificity 2 was set for any amino acid (ACDEFGHIKLMNPQRSTVWY); position in peptide 2 was anywhere, and protein N-term 2 and protein C-term 2 were selected. To search for cross-linked peptides, the cross-linker NMD (non-cleavable) was selected, minimum length for a paired sequence was set to 3, and the maximum peptide mass was set to 12 000 Da. The minimum peptide length for unspecified peptide search was 8 and maximum 25. The search included both intra-protein and inter-protein cross-linked peptides. Oxidation (M), acetyl (protein N-term), and carbamidomethyl (C) were included as variable modifications, with a maximum of 5 per peptide allowed. First search was performed with oxidation (M) and acetyl (protein N-term). Protein and cross-linked peptide level FDR was set to 1%. For each amino acid pair identified as cross-linked, the sum of the scores of all MS/MS entries evidencing the same pair was included in figure tables. The MaxQuant crosslinkMsms.txt files were filtered such that only amino acid pairs between ubiquitin C32 and an E3 protein that were evidenced with a sum of all peptide scores >100 were shortlisted. Specific filtering steps are described in Supplementary Datafile S1, but briefly, peptides defined by the following parameters were rejected from the list: (i) Decoy, (ii) Linear, (iii) Loop-linked, (iv) Mono + loop-linked, (v) Not cross-linked to C32 of ubiquitin, (vi) Not cross-linked to an E3 ligase, (vii) Cross-linked to a protein not expected in the sample, (viii) Cross-link from non-UV-irradiated samples, (ix) Non-E3 cross-link.

### Intact LC-MS

Intact LC-MS analysis was performed using 0.5 μg of protein injected in buffer containing 50 mM Tris, 150 mM NaCl, and 0.5 mM TCEP. LC-MS measurements were carried out on an Agilent 1200 LC-MS system equipped with a Max-Light Cartridge flow cell and coupled to a 6130 quadrupole mass spectrometer. An Agilent ZORBAX 300SB-C3 column (5 μm, 2.1 × 150 mm) was used unless otherwise stated. Protein elution was monitored by UV absorbance at 214 and 280 nm. Mass spectra were acquired in positive ion mode, and intact protein masses were determined by deconvolution using MS ChemStation software (Agilent Technologies).

### AlphaFold model generation

Structural models of Ub–E2–E3 complexes were generated by submission of protein sequences to AlphaFold [33]. To assess all identified cross-links, it was necessary to include the full constructs used in the cross-linking, including disordered domains. Alphafold model confidence scores (pTM and ipTM) are negatively affected by disordered domains. Nevertheless, all models had scores (>0.65) and appeared broadly consistent with expectation. In each case model ‘0’ was used in the analyses. All models and associated output data are included in Supplementary Datafile S2.

## Supplementary Material

Supplementary Figures S1-S6

Supplementary Data File S1

Supplementary Data File S2

## Data Availability

All AlphaFold models and associated data are included in Supplementary Datafile S2. The MS proteomics data have been deposited to the ProteomeXchange Consortium via the PRIDE partner repository with the dataset identifier PXD075613 [34].

## Open Access

Open access for this article was enabled by the participation of the University of Dundee in an all-inclusive Read & Publish agreement with Portland Press and the Biochemical Society under an agreement with JISC.

## Abbreviations

ACN: acetonitrile
ABP: activity-based probe
ABC: ammonium bicarbonate
AGC: automatic gain control
CV: Column Volumes
ERC: European Research Council
IMI2: Innovative Medicines Initiative 2
LB: Luria–Bertani
MS: mass spectrometry
NMD: N-maleimido diazirine
RING: Really Interesting New Gene
RV: rehydration volume
TFA: trifluoroacetic acid
Ub: ubiquitin
