# Supplementary material for: Photocross-linking activity-based probes to capture the dynamics of ubiquitin RING E3 ligase interactions
Source: Biochem J. 2026 Jun 3;483(7):1115–30. doi: 10.1042/BCJ20260213 (PMC13234630; doi:10.1042/BCJ20260213)
Supplement: Supplementary Figures S1-S6 [file BCJ-2026-0213_supp.pdf]

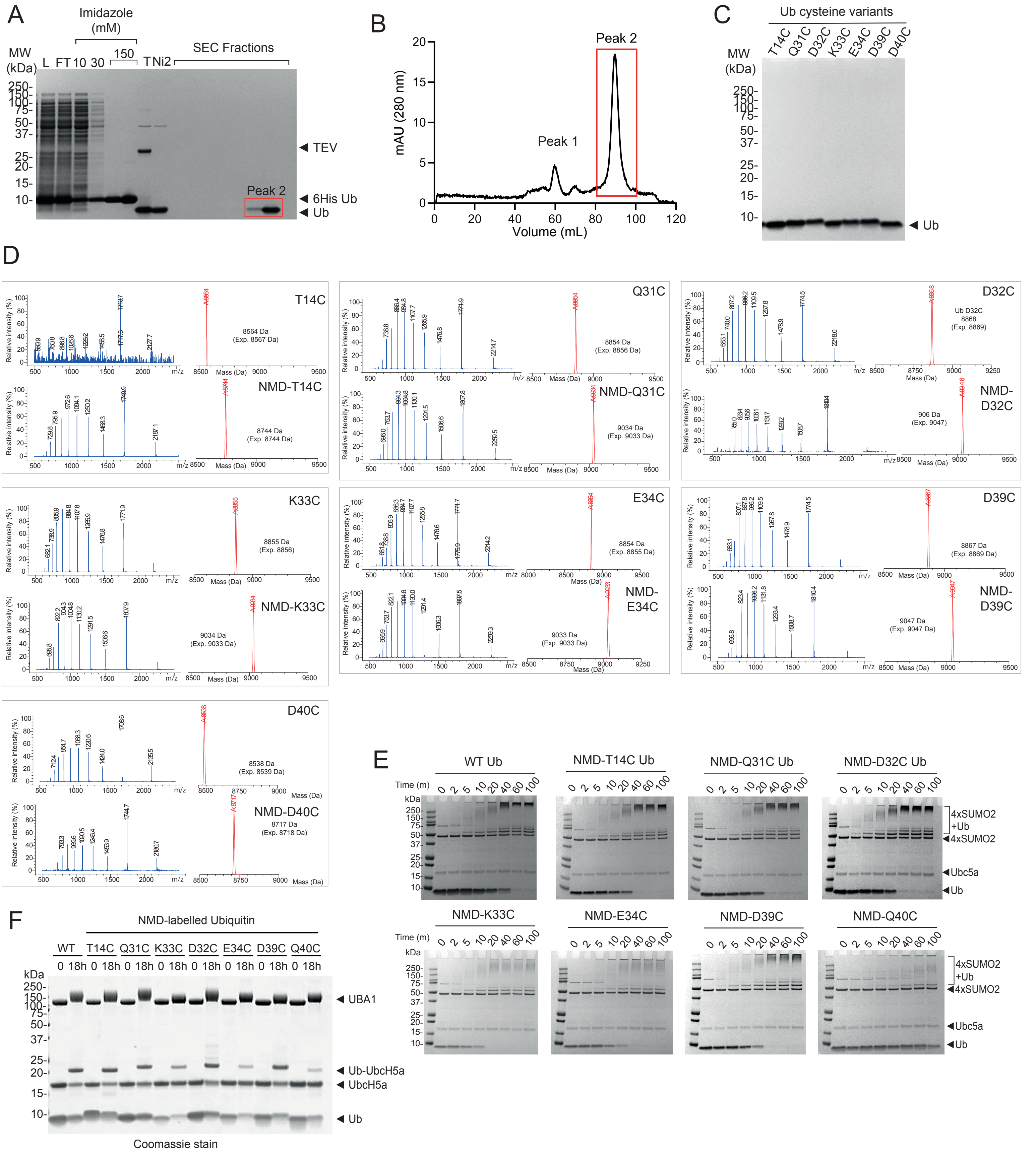

Supplementary Figure 1. Purification, NMD labelling and conjugation to UbcH5a of seven ubiquitin mutants. Related to Figure 1. A. Coomassie stained SDS-PAGE gel of protein samples taken during recombinant ubiquitin protein expression and purification (L = Nickel-NTA column load, FT – Nickel-NTA column flow through, 10 – 10mM imidazole wash, 30 – 30mM Imidazole wash, 150 – 150mM imidazole elutions, T – Purified 6His-Ub + TEV, Ni2 – Post TEV Nickel-NTA column flow-through, SEC – size exclusion chromatography. B. 280nm absorbance trace of SEC elution. C. Coomassie stained SDS-PAGE gel of 2 µg each purified cysteine ubiquitin variant. D. LC-MS for unlabelled and NMD-labelled ubiquitin variants as indicated. E. Conjugation of NMD-Ub variants (20 µM) to 4xSUMO-2 (5.5 µM) in the presence of RNF4 (0.55 µM), UbcH5a (0.5 µM), and UBA1 (0.1 µM). Samples taken after the indicated time points post ATP addition were analysed by Coomassie-stained SDS-PAGE. F. Conjugation of WT ubiquitin and NMD-Ub variants by UBA1 to UbcH5a C85K to form the NMD-Ub-UbcH5a ABPs.

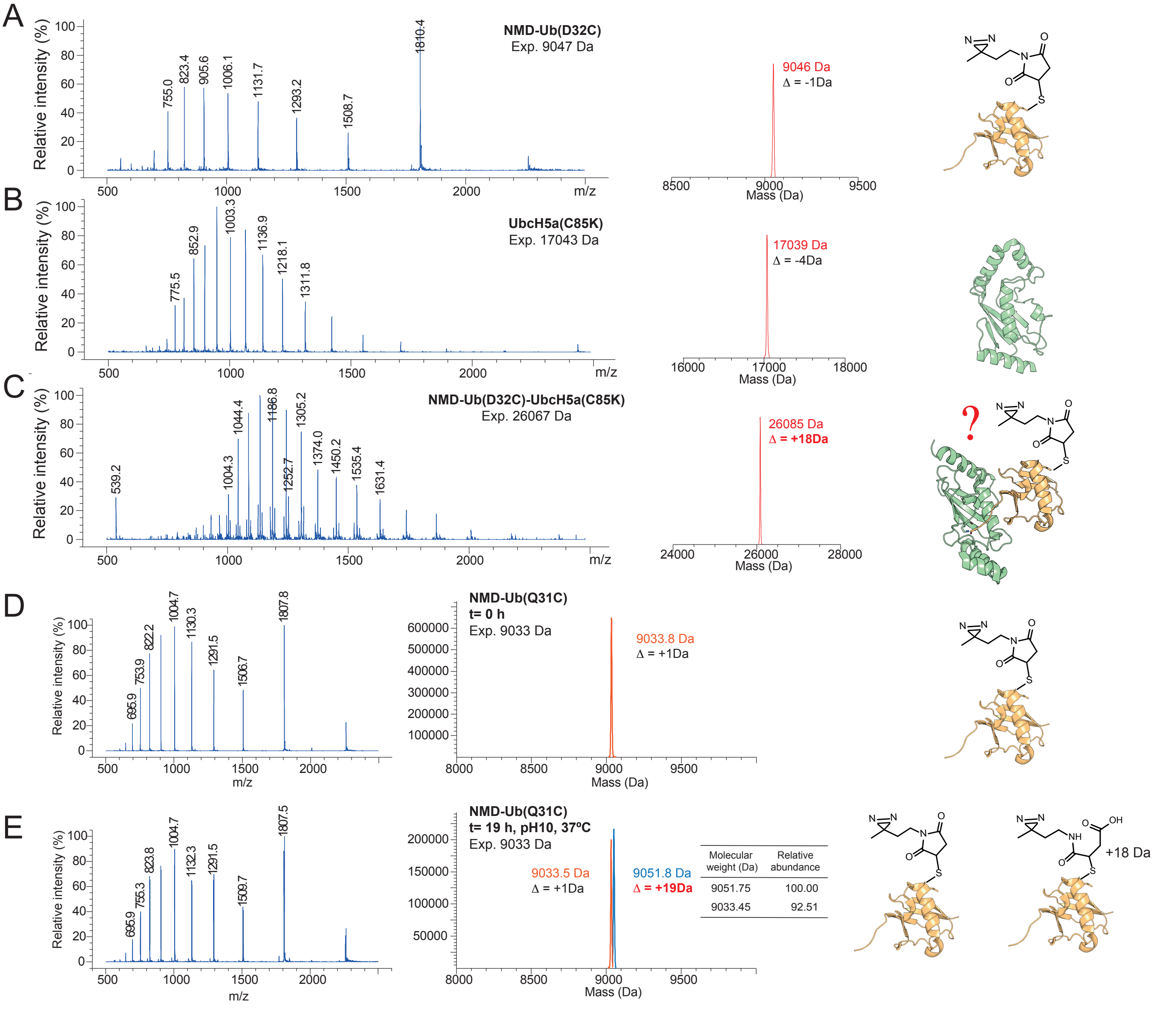

Supplementary Figure 2. Hydrolysis of NMD under conjugation assay conditions results in an 18 Da increase in mass of NMD-Ubiquitin. Related to Figure 2.
A-C. LC-MS analysis of NMD-labelled ubiquitin (D32C) (A), UbcH5a (C85K) (B) and NMD-Ub-UbcH5a (C). The expected mass for NMD-Ub-UbcH5a accounts for the loss of 18 Da during the formation of the isopeptide bond between Ub G76 and UbcH5a C85K. D+E. LC-MS analysis of NMD-Ub(Q31C) before (D) and after (E) incubation under the conditions of the ubiquitin conjugation assay.

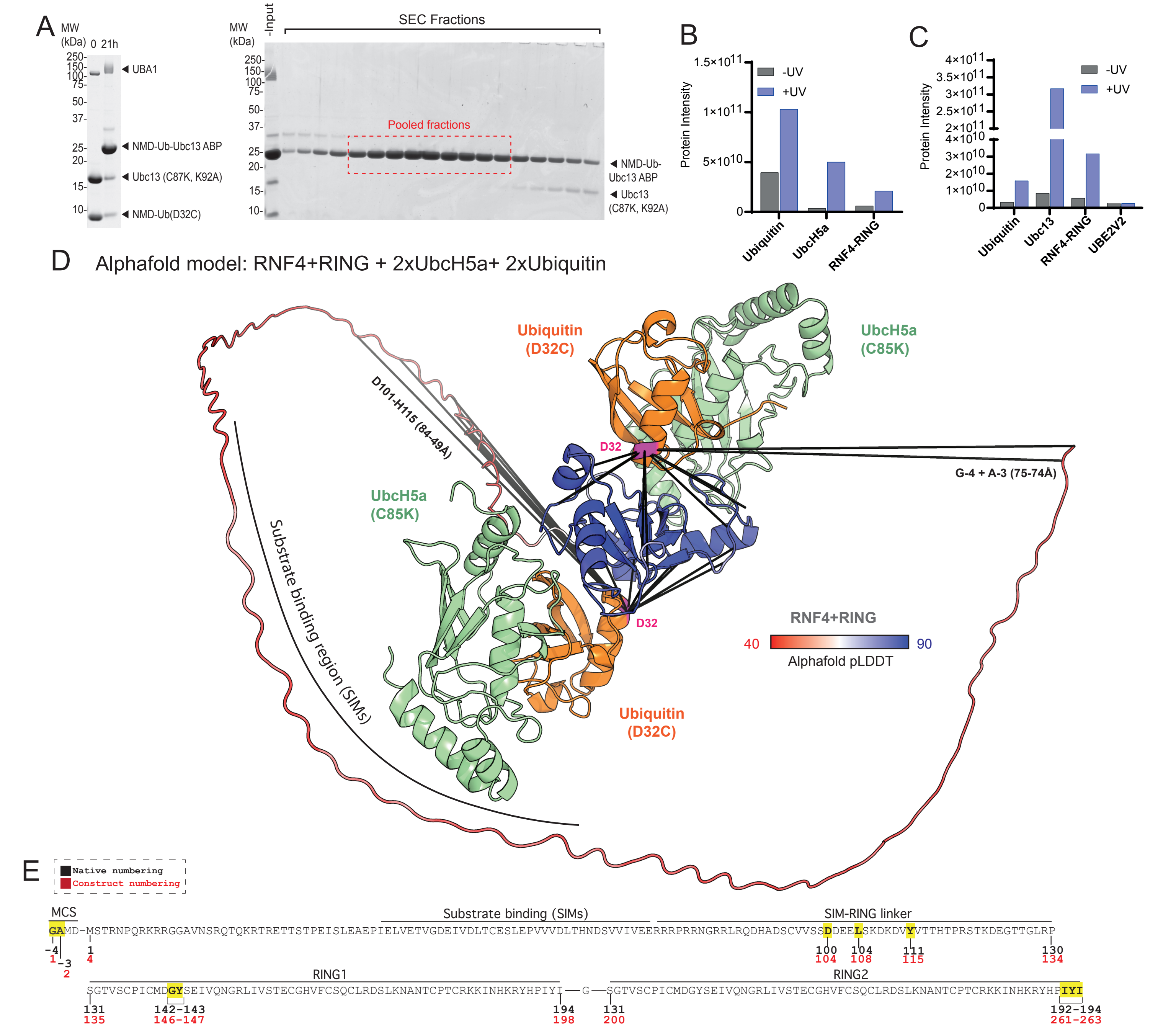

F

Residue	Position in RNF4-RING construct	Equivalent position in rnRNF4	Sum XL peptide Scores	Cα-Cα distance in 4AP4 (Å)
Gly	1	-4	397	NIC
Ala	2	-3	450	NIC
Asp	104	100	133	NIC
Leu	108	104	187	NIC
Tyr	115	111	308	NIC
Gly	146	142	147	19.40
Tyr	147	143	759	15.90
Ile	261	192	256	10.40
Tyr	262	193	1335	8.90
Ile	263	194	1796	11.90

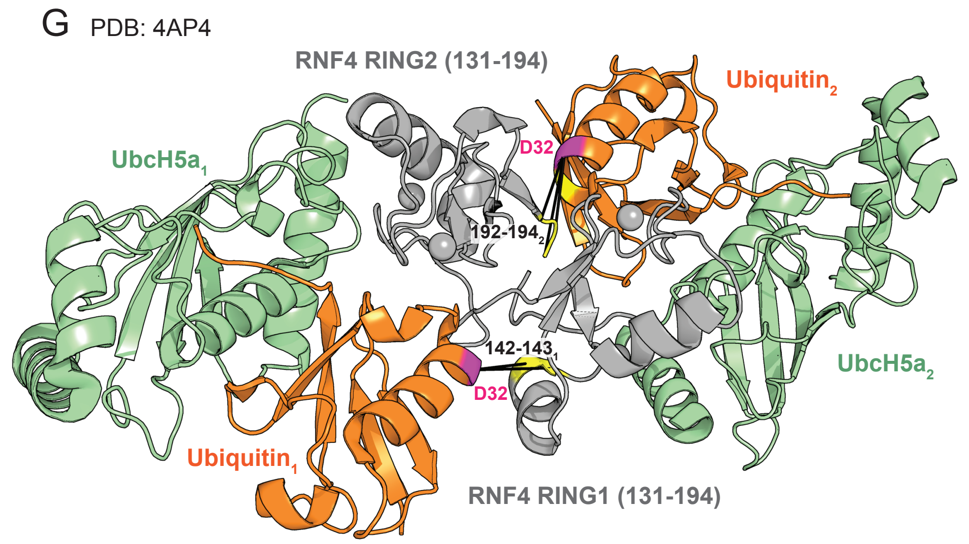

Supplementary Figure 3. UV-dependent high molecular weight adducts are indicative of crosslinking of NMD-Ub-Ubch5a and NMD-Ub-Ubc13 to RNF4+RING. Related to Figure 3. A. Conjugation of NMD-Ub to Ubc13 with samples taken prior to ATP addition ("0") or after 21 hours incubation at 37°C were analysed by SDS-PAGE (left). NMD-Ub-Ubc13 was purified by superdex 75 SEC (right) and pooled fractions indicated. B+C. Total peptide (protein) intensity values (sum of all non-crosslinked peptide intensities) for the indicated proteins in UV-specific species excised from the gels shown in Figure 2C and Figure 2D respectively. D. Alphafold model generated from hRNF4(1-190) and RNF4+RING(127-190) with two copies each of UbcH5a-C85K) (pale green) and ubiquitin (D32C) (orange). The RNF4 construct is coloured by pLDDT and long-distance crosslinks annotated. E. Sites of crosslinking to the RNF4+RING construct using the NMD-Ub-UbcH5a ABP. Distances according to the UbcH5a-RNF4+RING structure (PDB: 4AP4) are summarised in F and shown in G. NIC – Not in the construct used for the structural study. NM – Present in the construct used in the study but not modelled in the final structure.

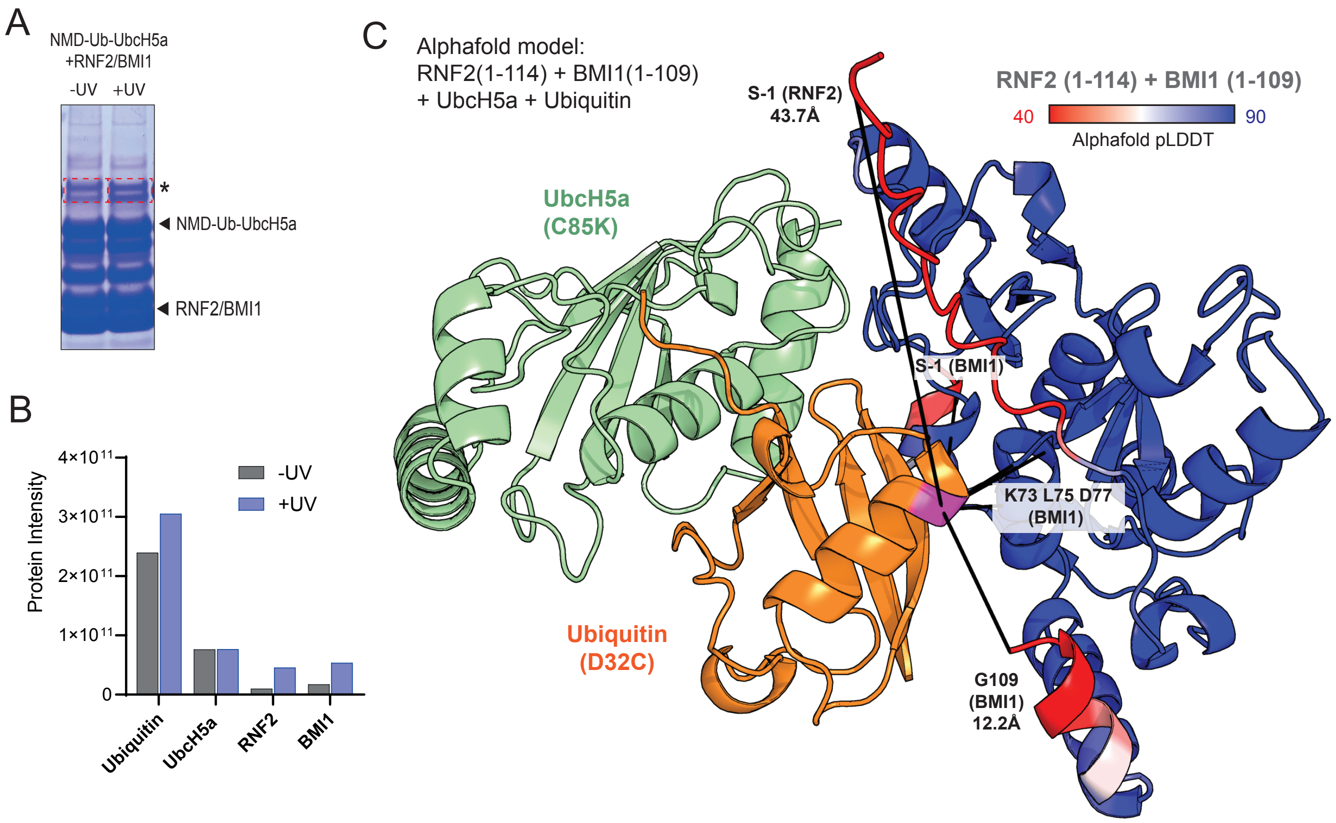

Supplementary Figure 4. Crosslinking of NMD-Ub-UbcH5a to RNF2/BMI1. Related to Figure 4.
A. SDS-PAGE analysis of UV irradiated (+UV) or non-irradiated (-UV) reactions containing RNF2(1-114)/BMI1(1-109) with NMD-Ub-UbcH5a. Identified regions (red box) were excised for digestion and LC-MS/MS analysis. *Asterisk indicates expected position for the cross-linked species. B. Total peptide (protein) intensity for the indicated proteins determined from non-crosslinked peptides determined by LC-MS/MS analysis of samples from A. C. Alphafold model of UbcH5a(C85K), Ubiquitin(D32C) (orange) and RNF2(1-114)/BMI1(1-109) coloured by pLDDT scores. Identified crosslinks are indicated.

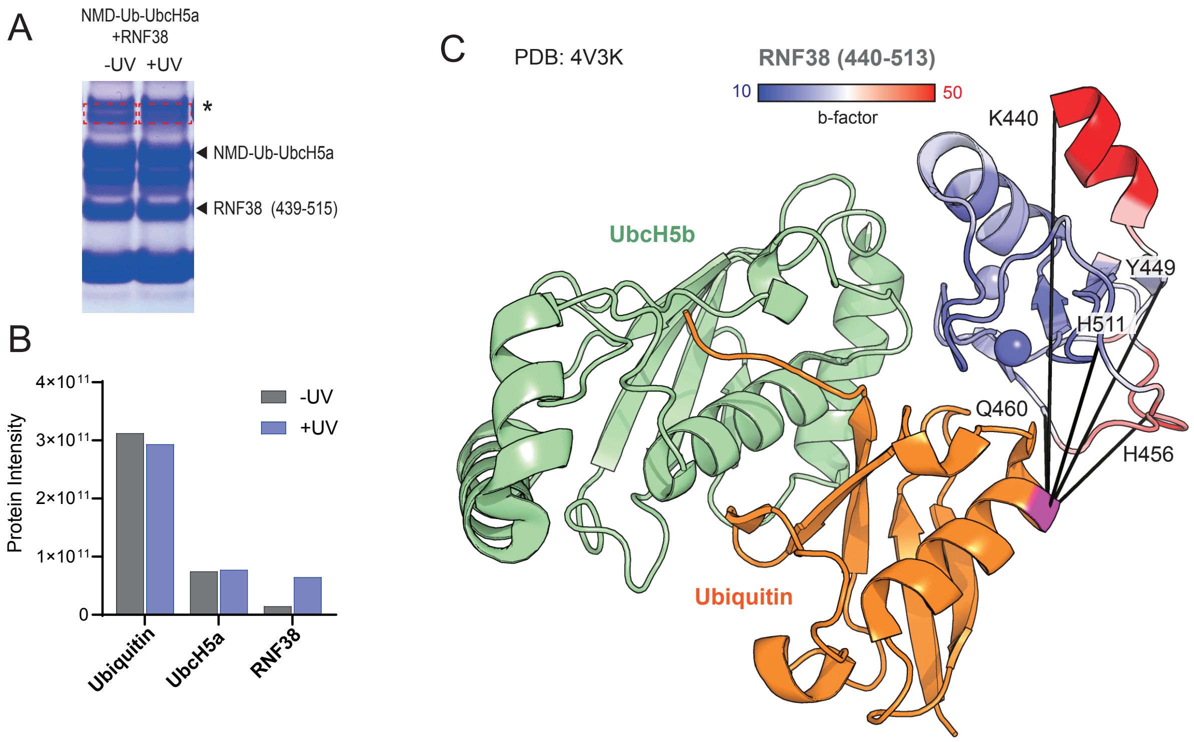

Supplementary Figure 5. Crosslinking of NMD-Ub-UbcH5a to RNF38. Related to Figure 5. A. SDS-PAGE analysis of UV irradiated (+UV) or non-irradiated (-UV) reactions containing RNF38(439-515) and NMD-Ub-UbcH5a. Identified regions (red box) were excised for analysis by LC-MS/MS. *Asterisk indicates expected position of crosslinked species. B. Total peptide (protein) intensity for the indicated proteins as determined by LC-MS/MS. C. Crosslinks from ubiquitin residue 32 to RNF38(440-513) in the structure PDB 4V3K. b-factor values are colour coded as in the key.

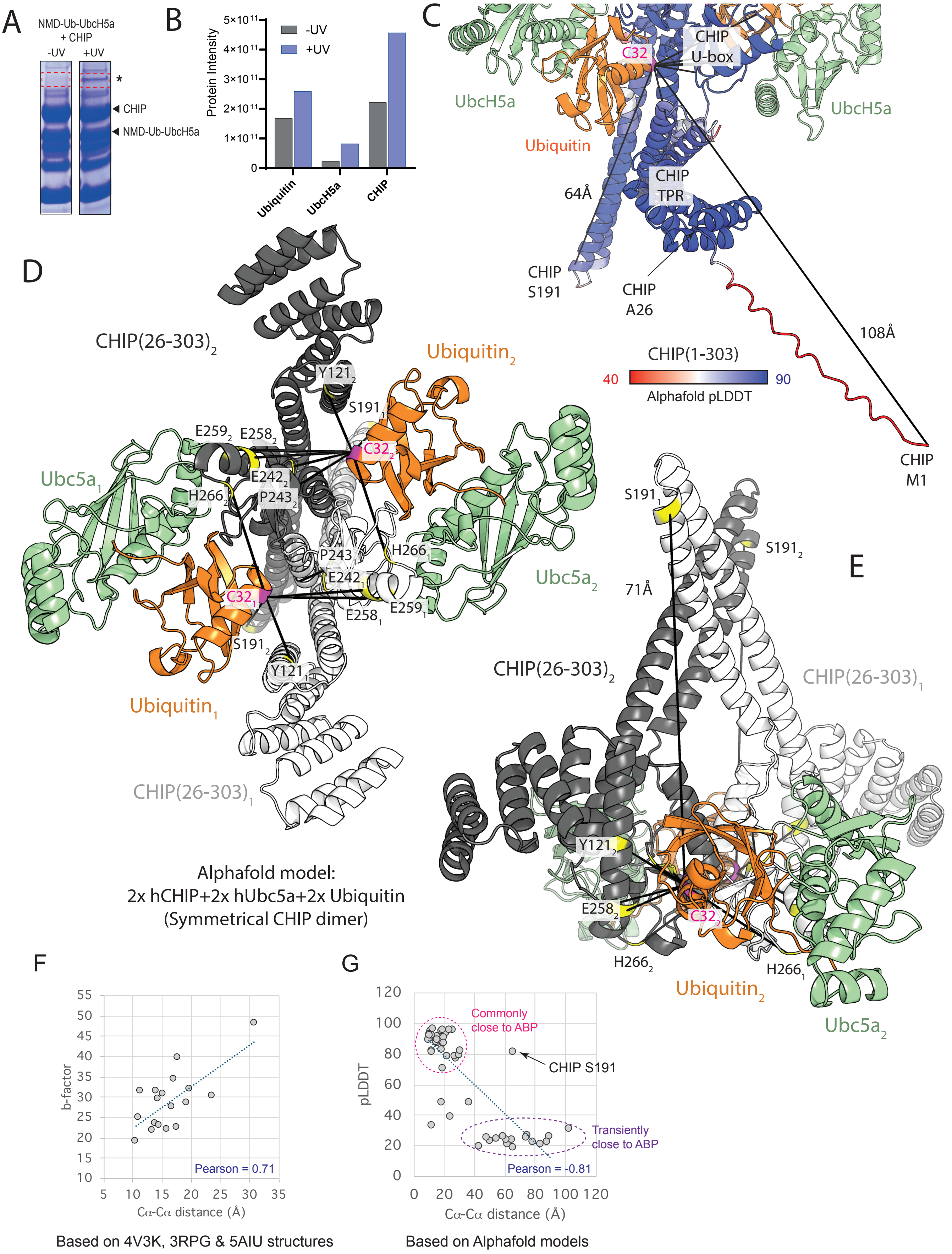

Supplementary Figure 6. A symmetrical dimer model for CHIP binding Ub-E2. Related to figure 6. A. SDS-PAGE analysis of UV irradiated (+UV) or non-irradiated (-UV) reactions containing full-length human CHIP with NMD-Ub-UbcH5a. Identified regions (red box) were excised from both lanes for digestion and LC-MS/MS analysis. *Asterisk indicated UV-dependent species. B. Total peptide (protein) intensity for the indicated proteins determined from non-crosslinked peptides determined by LC-MS/MS analysis of samples from A. C. Alphafold model of UbcH5a(C85K) (pale green), Ubiquitin(D32C) (orange) and two copies of full length human CHIP (coloured by pLDDT scores). Identified crosslinks are indicated. D, E. Alphafold model for a 2xUbcH5a, 2xubiquitin, 2xhCHIP(26-303) symmetrical dimer. Identified crosslinks are indicated. D is viewed along the axis of symmetry with the helical bundles projecting into the page, and E shows a side-view with one Ubiquitin-UbcH5a complex in the foreground. F. Relationship between measured crosslinker C -C distance and b-factor of the E3 ligase residue according to the structures shown in Figures 2J, 3D and 3H/S4F. G. Relationship between measured crosslinker C -C distance and Alphafold pLDDT values of the E3 ligase residues according to the models shown in Figures 4E, S3D and S4C. Pearson correlation coefficients are indicated in F and G.

SUPPLEMENTARY FIGURE AND FILE LEGENDS

Supplementary Datafile 1.

Summary of N-maleimido diazirine-derived crosslinked peptides reported by MaxQuant from the experiments described in this study. Unfiltered MaxQuant crosslinkMsms.txt files containing all identified crosslinks and final filtered lists are included.

Supplementary Datafile 2.

Alphafold models and associated data described in this study.

Supplementary Figure 1. Purification, NMD labelling and conjugation to UbcH5a of seven ubiquitin mutants. Related to Figure 1.

A. Coomassie stained SDS-PAGE gel of protein samples taken during recombinant ubiquitin protein expression and purification (L = Nickel-NTA column load, FT – Nickel-NTA column flow through, 10 – 10mM imidazole wash, 30 – 30mM Imidazole wash, 150 – 150mM imidazole elutions, T – Purified 6His-Ub + TEV, Ni2 – Post TEV Nickel-NTA column flow-through, SEC – size exclusion chromatography. B. 280nm absorbance trace of SEC elution. C. Coomassie stained SDS-PAGE gel of 2 µg each purified cysteine ubiquitin variant. D. LC-MS for unlabelled and NMD-labelled ubiquitin variants as indicated. E. Conjugation of NMD-Ub variants (20 µM) to 4xSUMO-2 (5.5 µM) in the presence of RNF4 (0.55 µM), UbcH5a (0.5 µM), and UBA1 (0.1 µM). Samples taken after the indicated time points post ATP addition were analysed by Coomassie-stained SDS-PAGE. F. Conjugation of WT ubiquitin and NMD-Ub variants by UBA1 to UbcH5a C85K to form the NMD-Ub-UbcH5a ABPs.

Supplementary Figure 2. Hydrolysis of NMD under conjugation assay conditions results in an 18 Da increase in mass of NMD-Ubiquitin. Related to Figure 2.

A-C. LC-MS analysis of NMD-labelled ubiquitin (D32C) (A), UbcH5a (C85K) (B) and NMD-Ub-UbcH5a (C). The expected mass for NMD-Ub-UbcH5a accounts for the loss of 18 Da during the formation of the isopeptide bond between Ub G76 and UbcH5a C85K. D+E. LC-MS analysis of NMD-Ub(Q31C) before (D) and after (E) incubation under the conditions of the ubiquitin conjugation assay.

Supplementary Figure 3. UV-dependent high molecular weight adducts are indicative of crosslinking of NMD-Ub-Ubch5a and NMD-Ub-Ubc13 to RNF4+RING. Related to Figure 3.

A. Conjugation of NMD-Ub to Ubc13 with samples taken prior to ATP addition ("0") or after 21 hours incubation at 37°C were analysed by SDS-PAGE (left). NMD-Ub-Ubc13 was purified by superdex 75 SEC (right) and pooled fractions indicated. B+C. Total peptide (protein) intensity values (sum of all non-crosslinked peptide intensities) for the indicated proteins in UV-specific species excised from the gels shown in Figure 2C and Figure 2D respectively. D. Alphafold model generated from hRNF4(1-190) and RNF4+RING(127-190) with two copies each of UbcH5a-C85K) (pale green) and ubiquitin (D32C) (orange). The RNF4 construct is coloured by pLDDT and long-distance crosslinks annotated. E. Sites of crosslinking to the RNF4+RING construct using the NMD-Ub-UbcH5a ABP. Distances according to the UbcH5a-RNF4+RING structure (PDB: 4AP4) are summarised in F and shown in G. NIC – Not in the construct used for the structural study. NM – Present in the construct used in the study but not modelled in the final structure.

Supplementary Figure 4. Crosslinking of NMD-Ub-UbcH5a to RNF2/BMI1. Related to Figure 4.

A. SDS-PAGE analysis of UV irradiated (+UV) or non-irradiated (-UV) reactions containing RNF2(1-114)/BMI1(1-109) with NMD-Ub-UbcH5a. Identified regions (red box) were excised for digestion and LC-MS/MS analysis. *Asterisk indicates expected position for the cross-linked species. B. Total peptide (protein) intensity for the indicated proteins determined from non-crosslinked peptides determined by LC-MS/MS analysis of samples from A. C. Alphafold model of UbcH5a(C85K), Ubiquitin(D32C) (orange) and RNF2(1-114)/BMI1(1-109) coloured by pLDDT scores. Identified crosslinks are indicated.

Supplementary Figure 5. Crosslinking of NMD-Ub-UbcH5a to RNF38. Related to Figure 5.

A. SDS-PAGE analysis of UV irradiated (+UV) or non-irradiated (-UV) reactions containing RNF38(439-515) and NMD-Ub-UbcH5a. Identified regions (red box) were excised for analysis by LC-MS/MS. *Asterisk indicates expected position of crosslinked species. B. Total peptide (protein) intensity for the indicated proteins as

determined by LC-MS/MS. C. Crosslinks from ubiquitin residue 32 to RNF38(440-513) in the structure PDB 4V3K. b-factor values are colour coded as in the key.

Supplementary Figure 6. A symmetrical dimer model for CHIP binding Ub-E2. Related to figure 6.

A. SDS-PAGE analysis of UV irradiated (+UV) or non-irradiated (-UV) reactions containing full-length human CHIP with NMD-Ub-UbcH5a. Identified regions (red box) were excised from both lanes for digestion and LC-MS/MS analysis. *Asterisk indicated UV-dependent species. B. Total peptide (protein) intensity for the indicated proteins determined from non-crosslinked peptides determined by LC-MS/MS analysis of samples from A. C. Alphafold model of UbcH5a(C85K) (pale green), Ubiquitin(D32C) (orange) and two copies of full length human CHIP (coloured by pLDDT scores). Identified crosslinks are indicated. D, E. Alphafold model for a 2xUbcH5a, 2xubiquitin, 2xhCHIP(26-303) symmetrical dimer. Identified crosslinks are indicated. D is viewed along the axis of symmetry with the helical bundles projecting into the page, and E shows a side-view with one Ubiquitin-UbcH5a complex in the foreground. F. Relationship between measured crosslinker $C\alpha$-$C\alpha$ distance and b-factor of the E3 ligase residue according to the structures shown in Figures 2J, 3D and 3H/S4F. G. Relationship between measured crosslinker $C\alpha$-$C\alpha$ distance and Alphafold pLDDT values of the E3 ligase residues according to the models shown in Figures 4E, S3D and S4C. Pearson correlation coefficients are indicated in F and G.
